# Training periodization for a world-class 400 meters individual medley swimmer

**DOI:** 10.5114/biolsport.2022.109954

**Published:** 2021-11-10

**Authors:** José María González-Ravé, David B. Pyne, José Antonio del Castillo, Fernando González-Mohíno, Michael H Stone

**Affiliations:** 1 Sport Training Laboratory. Faculty of Sport Sciences. University of Castilla la Mancha, Spain; 2 University of Canberra, Research Institute for Sport and Exercise, Faculty of Health, 11 Kirinari St, Bruce, ACT, 2617, Australia; 3 Facultad de Ciencias de la Vida y de la Naturaleza, Universidad Nebrija, Calle del Hostal s/n, 28248 Hoyo de Manzanares, Madrid, Spain; 4 Center of Excellence for Sport Science and Coach Education, Dept. of Kinesiology, Leisure and Sport Sciences, East Tennessee State University, USA; 5 Spanish Swimming Federation, Spain

**Keywords:** Progression, Male, Elite, Season, Macrocycles

## Abstract

We present a case study of the periodized training by a world-class 400-m Individual Medley (IM) swimmer (4^th^ in 2019 World Championships) in the season leading to a bronze medal in the 2018 European Championship. The complexity of this IM preparation was based on the experiences, observations and innovations of an Olympic swimming coach. Over 52 weeks, a traditional periodization model was employed using three macrocycles. A total of 15 competitions were completed in the season increasing in frequency in the third macrocycle. The training intensity distribution (TID) followed the pattern of a traditional pyramidal model in general training and polarized and threshold models during specific training before competitions. Weekly training volume ranged from 25 to 79 km, 24 to 87 km, and 25 to 90 km in each of the three macrocyles. Altitude training comprised 23% of total training weeks. Haemoglobin [Hb] increased from 14.9 to 16.0 g/100 ml and haematocrit from 45.1 to 48.1% after altitude training. Heart rate (HR) and [La^-^] decreased at submaximal swimming intensities, while swimming velocity increased in the 8 × 100 m incremental swimming test in A2 (1.4%) and in AT (0.6%). Pull up power was increased 10% through the season.

## INTRODUCTION

The individual medley (IM) events in swimming are performed with all four of the major strokes: freestyle, backstroke, breaststroke and butterfly. The IM is a more complex event than others because the training of four different strokes creates unique energetic requirements [1]. Many competitive swimmers spend most of their training time aiming to improve aerobic endurance, defined as the ability to sustain a high percentage of VO_2max_ for a long period, through careful and repeated activation of aerobic metabolism. This type of training is important for performance in events around 4 minutes such as the 400 m IM [2]. Accordingly, the physiological preparation for a 400 m IM should cover primarily maximal aerobic power (rate of adenosine triphosphate resynthesis), capacity (total amount of adenosine triphosphate resynthesis from available fuels) and VO_2max_ (maximum oxygen uptake) [1].

Numerous studies on swimming periodization have described a traditional model of training periodization [3–4], but there is little information on IM training. The traditional periodization was developed by Matveyev [5] for improving the sport performance in Soviet elite athletes. Each cycle was divided into preparatory, competitive and transition following the Matveyev’s proposal, with the aim of building aerobic capacity first through a period of high-volume/low-intensity training, before reducing volume and increasing the proportion of high-intensity training. The case study of a world-class athlete can provide unique insights into the training preparation [6]. The aim of this case study was to contextualize individual medley periodization as a complex combination of training the four strokes, across three training macrocycles in one season, including regular altitude training. We also evaluated the utility of a progressive incremental swimming test and selected power and biomedical tests to monitor changes.

## MATERIALS AND METHODS

The athlete described in this case study was a male international 400-m IM swimmer, Joan Lluís Pons Ramón (ESP), a finalist at the 2016 Rio Olympics and bronze medalist in 2018 European Championships. The swimmer joined the training program in 2014–15 reaching a performance standard between 850–900 FINA points in the 200 m butterfly and 400-m IM. The previous history of training showed an increase of annual training volume (from 2500 km in 2015 to 3300 km in 2018), the number of weeks in each season (from 46 to 52) and days of altitude training (from 40 in 2015 to 63 in 2018), and volume of altitude training (from 421 km in 2015 to 760 km in 2018). The athlete was 19 years old when he achieved a finals position at the 2016 Rio Olympics and a national record, and 21 years old at the Glasgow 2018 European Championships. The study was performed in accordance with the Principles of the Declaration of Helsinki and approved by the local ethics committee (Approval Number CSEULS-PI-114/2016). The athlete provided formal written approval for explicit publication of his name, performances and physiological details.

**FIG. 1:**
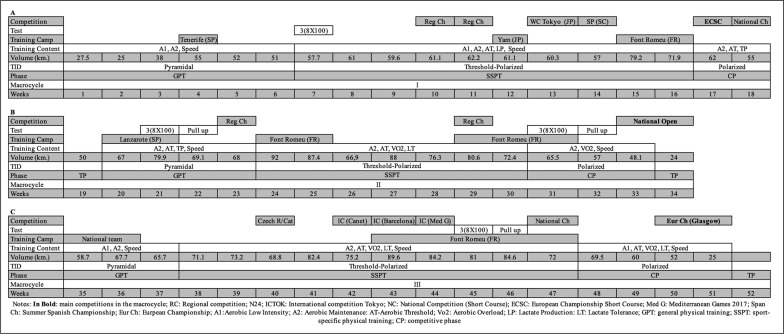
52 weeks of training periodization for a world-class 400 m-IM with three distinctive major competitions.

Figure 1 shows the main features of the season described in this study. The traditional periodization model was designed using three macrocycles, and each macrocycle divided in the preparatory phase with 2 sub-phases: general physical training and sport-specific physical training [7]. The competitive phase is when the athletes need to peak for a competition. Athletes may engage in mono-, bi- or tri-phasic periodized programs depending on the priority of competitions within a given year [8]. Three distinctive peaks of total load were identified, the main aim of the first macrocycle was to develop general physical fitness and specific qualities oriented to medley swimming. The goal of second and third macrocycles was developing specific qualities required for the 400-m IM, building from general to sport-specific qualities culminating in the taper and competition (Figure 1). The importance of each swimming session was coded from 1 (low) to 5 (high) over the season (Table 1). Figure 1 details the volume, training intensity distribution, training contents, training camp, competitions and tests in each macrocycle. The weekly maximum volume increased through the season (from 79 km to 90 km). There were marked increases in the weekly training volume between the first (55 ± 14 km) and second (68 ± 17 km) macrocycles, and between the first and third (69 ± 15 km) macro-cycles. Training intensity distribution was described with a five-zone system Z1–Z5 as Z1 =< 2 mmol/l; Z2 = from 2 to 4 mmol/l; Z3 => 4–6 mmol/l; Z4 = above 6 mmol/l of blood lactate concentration; and Z5 = maximal swimming speed [3].

**TABLE 1:** Training contents detailing sequence and level of priority of training macrocycle for a world-class 400-m individual medley swimmer. The priority of training contents shifted from aerobic in the 1^st^ macrocycle, threshold in the 2^nd^ macrocycle, and VO_2_ and race in the 3^rd^ macrocycle prior to major competition.

	Training contents		1^st^ macrocycle	2^nd^ macrocycle	3^rd^ macrocycle
			Level of priority		
Z1	A1		4	3	3
	A2	Aerobic	4–5	4	3
Z2	AT		3–4	4–5	4–5
Z3	VO_2_		2	4	5
Z4	LP	Race Pace	3	2	2
	LT		2	4–5	5
Z5	Speed	Race Speed	5	4	4

Strength-Hypertrophy			4–5	2	-
Maximal strength			3	3	3
Power			3	4	4
Power endurance			-	4	5
Core stability – strength endurance			5	3	3
General physical development			4–5	3	2
Sport-specific physical development			-	4–5	4–5
Flexibility			5	5	5

Al-Aerobic Low Intensity (< 50 bpm) A2-Aerobic Maintenance (40–50 bpm) AT-Aerobic Threshold (30–40 bpm) VO_2_-Aerobic Overload (10–20 bpm) LP-Lactate Production (0–10 bpm) LT-Lactate Tolerance (0–10 bpm) Speed-Basic Speed ATP-CP. Key: 1 = low priority. 5 = high priority. *Heart rate (bpm) below of HRmax.

The duration of the general, specific and competitive in each of the mesocycles were planned as follows: first macrocycle 6, 10 and 5 weeks; second macrocycle 4, 7 and 10 weeks; and the third macrocycle 3, 10 and 3 weeks. The first macrocycle emphasized development of aerobic power. The second macrocycle aimed to increase both aerobic power and the anaerobic (or lactate) threshold characterized by training up to 50 km/week. The third macrocycle increased the technical and physical fitness leading to peak performance at the 2018 European Championships.

Traditional resistance training is widely used in many sports and in swimming typically involves conventional gym-based resistance training exercises [9]. In the first macrocycle, strength and conditioning training (gym sessions) was focused on strength-hypertrophy, maximal strength, and strength-metabolic conditioning workouts (sessions which involved cardiovascular interval training or circuit training consisting of 6–12 exercises performed for prescribed time periods with light loads) with a duration ranging from 50–80 minutes. Mid-section (or core training) training sessions were also performed to enhance stability and prevent injuries common in swimmers. In the second and third macrocycle, gym sessions were focused on maximum strength, power and power endurance with resistance exercises. In addition, strength-metabolic conditioning workouts were focused on exercises with a similar duration (4 min) to the 400 m IM event. We continued with mid-section training sessions as in the first cycle. In addition, we progressively transformed from strength-metabolic conditioning workouts to muscular endurance with exercises that also approximated the duration of the event (˜4 min). Light and moderate weights were used in every exercise (30–50% 1-RM).

A graded incremental swimming test was employed to measure cardiovascular (HR), metabolic ([La^-^]), and mechanical (stroke rate using the stopwatch function and stroke count) responses to increasing speeds of swimming. The protocol was as follows: 3 sets of 8 × 100 m freestyle with set 1 at A2 (moderate-intensity aerobic work ˜85% personal best (PB) time), 1’30” rest; set 2 at LT (lactate threshold 4 mmol L^-1^ velocity ˜90% of PB time), 1’40” rest; set 3 at MVO_2_ (maximal aerobic work ˜95% of PB time), 1`50” rest [10]. Tests were conducted in each macrocycle. Blood samples of 0.5 μl for lactate analysis (Lactate Scout, SensLab GmbH, Germany) were collected from a fingertip 30 s after each set of 8 × 100 m performed by the swimmer. The swimming velocities corresponding to 4 and 8 mmol L^-1^ (v 4 and v 8), proposed as standards for lactate threshold and aerobic power, were computed [11].

Regarding hematological data for evaluating the effects of altitude training, venous blood samples (4 ml) were drawn from an antecubital venipuncture early in the morning and 3 days before the altitude training camp, and after the first day returning to sea level. Blood samples were analyzed in duplicate for haemoglobin concentration (Radiometer OSM-3) and haematocrit (spun capillary tubes).

The gym-based testing involved five repetitions of pull ups with bodyweight according to the protocol of Coyne et al. [12] Power (w) and mean velocity (m · s^-1^) were measured using a Smartcoach ® encoder.

A descriptive analysis was performed, using means and percentage of change. To assess the changes of weekly volume per macro-cycle, a paired t-test was performed. Changes were interpreted against the smallest important difference in competition performance.[6] For the analyses, significance was set at p < .05.

## RESULTS

The swimmer achieved 5^th^ place in the European Championship in a 25 m pool (4:08:56) in December 2017, 1^st^ place in Open National Championship in April 2018 (4:18:10), 3^rd^ place in the 2018 European Championship (4:14:26), and a personal best time and 15^th^ place in the 2020 Tokyo Olympic Games (4:12.67)

In the 8 x 100 swimming test the mean swimming velocity was increased at A2 (1.3%) and LT (0.6%) intensities with no change in velocity at VO_2max_ through the season. Similarly [La^-^] at A2 (3.6%) and VO_2max_ (3.6%) and HR decreased at the same intensities indicating a good performance progression as shown in Table 2. Stroke rate increased slightly at A2 and MVO_2_, and stroke number decreased at A2 and MVO_2_ (Table 2).

**TABLE 2:** Results of 3 × (8 × 100 m) and Pull up tests showing marked improvements in performance, physiological measures, and upper body power. HR = heart rate, Vel. = velocity

3 × (8 × 100 m) test							Pull up test	
Date of test Macrocycle	Intensity	Lactate (mmol·L^-1^)	HR (bpm)	Stroke number	Stroke Rate	Velocity (m·s^-1^)	Vel.(m·s^-1^)	Power (w)
Oct-2017 (M1)	A2	1.9	162	32	1.34	1.47		
	AT	2.7	174	32	1.34	1.56	-	-
	MVO_2_	10.7	192	39	1.10	1.69		

Jan-2018 (M2)	A2	2.1	155	31	1.39	1.48		
	AT	2.6	172	33	1.30	1.56	0.79	502
	MVO_2_	8.2	186	39	1.10	1.68		

Mar-2018 (M2)	A2	1.9	160	31	1.39	1.49		
	AT	2.8	174	33	1.30	1.57	0.83	542
	MVO_2_	8.9	188	35	1.23	1.71		

Jun-2018 (M3)	A2	1.8	156	31	1.39	1.49		
	AT	2.8	174	33	1.30	1.57	0.88	586
	MVO_2_	6.8	182	35	1.23	1.69		

M: Macrocycle; A2: moderate-intensity aerobic work; A3: AT-Aerobic Threshold; MVO_2_: Maximal aerobic work.

Through the season, the swimmer completed four altitude training camps (Figure 1). Altitude training represented a substantial percentage of the season (23% of total weeks) to promote hemato-logical adaptations and performance during the subsequent training and/or competition period. At the beginning of the first macrocycle (week 13), the swimmer had values of Hb of 15.9 g/100 ml and 46.8% of hematocrit. At the end of the first macrocycle (week 17), he participated in an altitude training camp (Font Romeu, France 1,850 m) over 10 days with values of Hb of 15.9 g/100 ml and 47.2% of hematocrit (an increase of 0.8% in absolute hematocrit). In the second macrocycle, he completed two altitude training camps (14 and 15 days, respectively), during the specific and competition periods. Values were stable at 15.3–15.4 g/100 ml of Hb and 45.3–45.5% of haematocrit (weeks 26 and 32). Finally, during the 28-day altitude training camp in the third macrocycle, Hb increased from 14.9 to 16.0 g/100 ml, and the haematocrit from 45.1 to 48.1% (from week 42 to week 48).

Finally, mean velocity and power were increased in the pull ups test through the season. Power increased by 7.4%, and mean velocity by 4.8% over 3 months of the second macrocycle. In the next macrocycle, the power increased by 7.6% and mean velocity increased by 5.7% from 0.83 to 0.88 m · s^-1^ as shown in Table 2.

## DISCUSSION

The traditional periodization model employed for this high-level swimmer was based on 3 macrocycles to achieve the peak performance at the major international competition (Glasgow 2018 European Championships). An individualised approach was a key feature for developing the hierarchy of training contents (Table 1). The results achieved in competition confirmed that the plan produced peak performance at the appropriate times. Manipulation of training volume and intensity yielded physiological, hematological and performance adaptations via an overcompensation process [9]. The season was divided into 3 macrocycles, different to previous studies that reported 1 or 2 macrocycles for elite swimmers. For example, the retrospective study of Hellard et al. [3] conducted on 127 elite swimmers and 20 competitive seasons, characterised training into 4 mesocycles (in the case of 1 macrocycle) or 4–6 in the case of 2 macrocycles. This option seems to be the most common among coaches [13–14]. In contrast, we divided every macrocycle into 3 mesocycles each (9 mesocycles in total) for a finer prescription of training.

The phases of each macrocycle are in contrast to other descriptive studies [3–4]. Throughout the development of the season, the specific and competitive phases were given more importance than the general preparation. A priority was for the swimmer to retain progressions from earlier macrocycles, providing sufficient background and stimuli to enhance targeted abilities. Regarding the endurance intensity zones, the rational sequencing and timing progressed from Z1 and Z2 in the first macrocycle, to Z3 and Z4 in the second and third macrocycles (Table 1). Maintaining a high level of priority and balance between Z2 and Z5 was also important, simulating the specific physiological requirement of this competitive event. The specific contribution of these zones depended on both the energetic contribution of 400-m race, and the pacing profile used in competition according to the energetic requirements for middle distance events [1].

The weekly volume performed in our study ranged from 25 to 79 km/week in the first macrocycle, from 24 to 87 km/week in the second macrocycle and from 25 to 90 km/week in the third macro-cycle. Other studies of elite swimmers reported weekly training volumes of approximately 55–60 km/week for the Italian national squad [11], while British sprint swimmers swam ˜43 km/week per week and long-distance swimmers ˜58 km/week [15]. A higher training volume could have contributed to improvements in technical swimming efficiency as a consequence of additional training [16].

The training intensity distribution in this case study followed a pattern of a traditional pyramidal model in general training, and a threshold-polarized model for specific training, and a polarized model prior to competition. This pattern of training intensity distribution was associated with improvements in testing of 3 × (8 × 100 m) through the season in which velocity increased at A2, LT with no improvements in MVO_2,_ as well as classical reductions in lactate and HR at submaximal intensities. Previous studies with elite swimmers showed a higher percentage of training (44–46%), in the 2–4 mmol · l^-l^ zone [17]. Pla et al. [18] reported greater improvements with polarized training compared to pyramidal training for 100 m performance. We agree with previous assertions that a possible explanation for this difference could be the higher technical swimming efficiency in the 2–4 mmol · l^-l^ zone training intensity zone [3]. Technical improvements probably account for the lower stroke rate at A2 and MVO_2_, and reduction in stroke count at A2 and MVO_2_ over the season.

The swimmer completed four different altitude training camps that ranged in duration between 10 and 28 days. Athletes should tailor repeated altitude exposures to emphasize the training goals of the macrocycle. Multiple altitude exposures during a season interspersed by prolonged periods longer than 8 weeks at sea level have been recommended [19, 20]. However, in the case of our swimmer, 6 weeks were interspersed between the first and second training camps, only three between the second and third, and 11 weeks between the third and fourth, given the proximity of important competitions. It should be noted there was only 3 weeks between the last altitude camp and the main competition of the season.

We monitored power and mean velocity in the pull up test with 4–8% improvements through macrocycles 2 and 3. Free-weight strength training (e.g. pull up exercise) have provided similar power improvements compared to weight-assisted training in swimmers [21]. Different dry-land exercises such as the lat pull-down, the bench press, or throwing a weighted medicine ball, can improve swimming power [15]. Low-volume, high-velocity/force resistance-training programmes resulted in significant improvements in swimming performance [9]. The mid-section core training performed across the season may have a significant role to play in swimming performance due to weaknesses within the lumbar and thoracic regions [9].

The swimmer’s progression continued according to the principles of overload and individualization in the following season achieving fourth place in the 2019 Swimming World Championships hosted in Gwangju (South Korea). According to Del Castillo et al [22]., to achieve better times in the 400-m competition, male swimmers should improve their time in the 200-m event, especially the back-stroke and the 400-m freestyle, and to a lesser extent, the 800- and 1500-m freestyle. A limitation of this study is the challenge of generalizing and transferring outcomes to other swimmers given the case study design. However, the case study is still a useful approach acknowledging of course the inherent limitations. A case study can be used to explain, describe, and explore events or phenomena in the everyday contexts in which they occur. In this study we have provided a unique insight into the training preparation of a world-class swimmer.

## CONCLUSIONS

Using a single-case approach, we have presented the novel training of an IM world-class male swimmer involving sequential manipulation of training cycles and training intensity. The order of training intensity models within each macrocycle was pyramidal (general training) to threshold-polarised (specific training) and finally polarized prior to major competition. The season was successful with substantial improvements in strength, fitness and competition performance. Regular monitoring of both training and competitive swimming performance, power and selected physiological measures informed coaching decisions.
